# Sexual behavior and its relationship with semen quality parameters in Sahiwal breeding bulls

**DOI:** 10.14202/vetworld.2015.745-749

**Published:** 2015-06-18

**Authors:** Shushant Singh, M. Bhakat, T. K. Mohanty, A. Kumar, A. K. Gupta, A. K. Chakravarty, P. Singh

**Affiliations:** 1 Artificial Breeding Research Centre, National Dairy Research Institute, Karnal - 132 001, Haryana, India; 2 Livestock and Production Management Division, National Dairy Research Institute, Karnal - 132 001, Haryana, India

**Keywords:** correlation, Sahiwal bulls, semen quality, sexual behavior

## Abstract

**Aim:**

The study was conducted at Artificial Breeding Research Centre, NDRI, Karnal, to determine the sexual behavior and its relationship with semen quality parameters in Sahiwal breeding bulls.

**Materials and Methods:**

A total of 63 ejaculates were collected from six adult Sahiwal bulls (age ~47 mo and bwt ~466 kg), to study the relationship of sexual behavior and semen quality. The degree of association between different variables was estimated by Pearson’s correlation coefficient method.

**Results:**

The results depicted that, sexual aggressiveness showed significantly high positive correlation with libido score (LS) and sexual behavior score (SBS). Reaction time (RT) and total time taken in mounts (TTTM) had a significant negative correlation with LS and SBS. Penile erection score and penile protrusion score (PPS) both had a significant positive correlation with ejaculatory thrust score, mating ability score, and SBS. Results of correlation among seminal attributes and with sexual behavior depicted that ejaculate volume had positive significant correlation with initial progressive motility (IPM), sperm concentration (SCON), head abnormality, total abnormality, hypo-osmotic swelling test (HOST), acrosomal integrity (AI) whereas, mass activity had positive significant correlation with IPM, SCON, non-eosinophilic spermatozoa count (NESC), HOST, AI, RT and TTTM and IPM had positive significant correlation with SCON, NESC, HOST, AI, and TTTM, whereas and HOST had positive significant correlation with AI. Among seminal attributes, SCON had a positive significant correlation with PPS where as head abnormalities had a positive significant correlation with RT and TTTM.

**Conclusion:**

It can be concluded that the relationship of sexual behavior and semen quality parameters are reflecting that the sexual behavior of individual bulls is important to harvest good quality and quantity of semen as desired type of sexual preparation can be provided.

## Introduction

Demand of frozen semen of elite Sahiwal bulls is increasing throughout the country due to its milk productivity, adaptability, disease resistance, heat tolerance, and survivability on poor feed and fodder resources. To achieve the optimum target of quality frozen semen production with better fertility breeding soundness evaluation (BSE) of bulls is important, for which sexual behavior and semen quality of breeding bulls need to be studied along with their relationship.

Studies of semen characteristics and correlation among various semen characteristics [1] of bulls are on records. Relationship between sexual behavior and semen quality reflects the reproductive efficiency of breeding bulls and has paramount importance in breeding program. To optimize the production performance of individual bulls, sexual behavior and semen quality can play an important role to decide suitable management interventions for quality semen production.

Selection of bulls on the basis of sexual behavior and semen quality are more important and economical [2]. In terms of prediction, if two variables are correlated perfectly, then knowing the value of one score permits a perfect prediction of the score on the second variable. However, literature is scanty on these aspects in adult breeding bulls for indigenous cattle bull. Therefore, the purpose of the present study was to observe the relationship among sexual behavior and various physico-morphological characteristics of semen to maximize the quality semen production and genetic improvement through optimum breeding and management strategies in adult Sahiwal bulls.

## Materials and Methods

### Ethical approval

The work was carried out under the institute project (no. B-33) entitled “Management practices to improve semen productivity by mitigating low libido problem in Sahiwal bulls. We have carried out experiment in accordance with the Guidelines laid down by the Institutional ethics committee of ICAR-NDRI with prior approval.

### Sample size and study location

Total of 63 ejaculates were collected from six Sahiwal bulls (body weight 390-540 kg; age 34-56 months) maintained under identical feeding and management condition during the experiment (19-03-2014 to 05-05-2014) at Artificial Breeding Research Centre, NDRI, Karnal. Healthy, sexually mature and clinically normal bulls showing good libido were selected randomly from the herd.

### Semen quality

Two successive ejaculates were collected from the bulls using sterilized bovine artificial vagina (IMV model-005417) maintained between 42-45°C, over a male dummy bull. Soon after collection, each ejaculate was placed in a water bath at 30°C and various standard laboratory tests for semen volume and mass activity (MA), individual progressive motility, sperm concentration (SCON) (million/ml) [3], non-eosinophilic spermatozoa count (NESC), hypo-osmotic swelling test (HOST) [4], acrosomal integrity (AI), and sperm abnormalities were recorded using differential interference contrast phase contrast microscope (Nikon Eclipse E600, Tokyo, Japan) with Tokoiheat thermal stage. The pH of the fresh semen was determined within 15 min of collection with Cyberscan 510 pH meter (EutechInstrument, Singapore).

### Sexual behavior

Sexual behavior like sexual aggressiveness (SA), reaction time (RT), dismounting time (DT), total time taken in mounts (TTTM), penile erection score (PES), penile protrusion score (PPS), ejaculatory thrust score (ETS), mating ability score (MAS), and sexual behavior score (SBS) was studied using CCTV video camera recording on the basis of different score card [2] with slight modification. SBS was divided into two parts; libido score (LS) and MAS. Libido was scored on the basis of RT (in seconds), SA, and tactile stimulation. Libido was scored on the basis of RT (in seconds), SA, and tactile stimulation. SA: SA was classified and scored (in bracket) as aggressive (4), active (3), dull and shy (1). Tactile stimulations (TS) comprises sniffing, flehmen reaction, licking, pawing, nudging, chin-resting, licking of penis, and urinating. RT, DT, and TTTM: RT is the time lapse between the appearance of bull to the dummy and its first mount or mounting (MO) attempt. The mount may or may not be results with successful ejaculation. DT is the time lapse between ejaculatory thrust (semen ejaculation) and stepping down of the front legs in the ground, i.e., activity followed after the intensity of thrust and ejaculation. TTTM is the duration of time taken by a non-stimulus male from the appearance to amount with successful ejaculation when it brought to a stimulus bull. RT (second) classified and scored (in bracket) as up to 10 (5), 11-30 (4), 31-60 (3), 61-120 (2), 121-300 (1), and above 300 (0). LS: For every TS expression 0.2 was deducted from the total score obtained from RT and SA. A LS (%) of each ejaculates was computed as shown below:

[{(RT score + SA score)−0.2 per TS}/10])×100

MAS: MAS was scored on a 10 point scale distributed among different behavioral events displayed by bulls during semen collection i.e. MO, PES, PPS, ETS, grasping of teaser at pelvic level (Gr), and penile movement to locate artificial vagina (Pm). After MO (except false mount) if a bull did not ejaculate, then 1 point was deducted from the total score for each futile attempt. The MAS was determined in successful attempts only as follows:

[{(MO+PES+PPS+ETS+Gr+Pm)-Futile attempts}/10)] ×100.

SBS: The SBS was calculated from the LS and MAS as follows:

(LS + MAS)÷2

### Statistical analysis

The degree of association among and between sexual behavior and seminal attributes were estimated by Pearson’s correlation coefficient method and significance was tested by Student’s *t-test* as suggested [5].

## Results and Discussion

The overall least squares means of ejaculate volume, MA, IPM, SCON pH, NESC, total morphological abnormality (head, mid-piece and tail), HOST, and AI were 3.40±0.20 ml, 2.90±0.11, 73.20±2.1%, 1130.10±38.45 million/ml, 6.80±0.04, 84.30±1.63%, 6.59±0.21% (2.34±0.23%, 0.79±0.09%, 3.59±0.16), 63.15±1.72%, 89.00±1.44%, respectively. The overall least squares means of SA, RT, DT, TTTM, PES, PPS, ETS, LS, MAS, SBS, were 3.75±0.06, 239.41±28.40 s, 4.41±0.21 s, 243.81±28.41 s, 2.97±0.02, 1.90±0.04, 2.62±0.07, 93.84±1.13%, 46.09±1.80%, 70.37±1.10%, respectively in Sahiwal breeding bulls.

The relationships of sexual behavior and semen quality have great significance to predict the quality and fertility of bulls. The results of correlation coefficients among different sexual behavior parameters, semen quality parameters and between them are presented in Tables-1-3.

**Table-1:** Correlation matrix showing coefficients of correlation among sexual behavior parameters of adult Sahiwal bulls.

Parameters	SA	RT	DT	TTTM	PES	PPS	ETS	MAS	LS	SBS
SA	1.00									
RT	−0.08	1.00								
DT	0.19	0.05	1.00							
TTTM	−0.08	0.99**	0.06	1.00						
PES	0.21	0.10	0.26*	0.10	1.00					
PPS	0.17	0.01	0.23*	0.02	0.39	1.00				
ETS	0.08	−0.23*	0.16	−0.23*	0.35**	0.71**	1.00			
MAS	0.13	−0.13	0.24*	−0.13	0.56**	0.82**	0.95**	1.00		
LS	0.46**	−0.56**	0.15	−0.56**	0.24*	0.08	0.20	0.21	1.00	
SBS	0.35**	−0.41**	0.25*	−0.40**	0.54**	0.64**	0.80**	0.84**	0.71**	1.00

SA=Sexual aggressiveness, RT=Reaction time, DT=Dismounting time, TTTM=Total time taken in mounts, PES=Penile erection score, PPS=Penile protrusion score, ETS=Ejaculatory thrust score, MAS=Mating ability score, LS=Libido score, SBS=Sexual behavior score.

** p<0.01;

* p<0.05

**Table-2:** Correlation matrix showing coefficients of correlation among seminal attributes of adult Sahiwal bulls.

Parameters	E.Volume	MA	IPM	pH	CON	NESC	Head	Mid-piece	Tail	Total	HOST	AI
Volume	1											
MA	0.22	1										
IPM	0.27*	0.79**	1									
pH	0.03	0.11	0.17	1								
CON	0.32**	0.58**	0.47**	0.03	1							
NESC	0.24	0.72**	0.93**	0.22	0.38**	1						
Head	0.30*	−0.05	−0.17	0.10	−0.04	−0.18	1					
Mid-piece	0.03	−0.13	−0.26*	−0.22	0.05	−0.22	0.31*	1				
Tail	0.01	−0.02	−0.07	−0.18	−0.07	−0.17	−0.12	0.30*	1			
Total	0.26*	−0.06	−0.22	−0.11	−0.01	−0.27*	0.76**	0.65**	0.48**	1		
HOST	0.27*	0.64**	0.80**	0.27*	0.46**	0.87**	−0.12	−0.13	−0.16	−0.20	1	
AI	0.27*	0.49**	0.73**	0.09	0.22	0.79**	−0.14	−0.08	0.08	−0.08	0.74**	1

MA=Mass activity, IPM=Initial progressive motility, CON=Sperm concentration (million/ml), NESC=Non-eosinophlic spermatozoa count, Head=Head abnormality, Tail=Tail abnormality, Total=Total abnormality, HOST=Hypo-osmotic swelling test, AI=Acrosomal integrity.

** p<0.01,

* p<0.05

**Table-3:** Correlation matrix showing coefficients of correlation between seminal attributes and sexual behavior parameters of adult Sahiwal bulls.

Parameters	SA	RT	DT	TTTM	PES	PPS	ETS	MAS	LS	SBS
E. Volume	−0.04	0.11	−0.02	0.11	−0.19	−0.24	−0.22	−0.25	0.04	−0.15
MA	0.12	−0.34**	0.13	−0.34*	−0.15	0.14	0.15	0.11	0.21	0.19
IPM	0.10	−0.28*	0.11	−0.28*	−0.09	0.02	0.04	0.03	0.20	0.13
pH	0.16	−0.08	−0.24	−0.08	−0.16	−0.08	−0.13	−0.17	−0.10	−0.18
CON	0.13	−0.10	0.08	−0.10	−0.009	0.28*	0.16	0.13	0.18	0.19
NESC	0.02	−0.19	0.09	−0.19	−0.13	−0.08	−0.05	−0.06	0.13	0.02
Head	0.22	0.36**	−0.18	0.36**	0.05	0.06	0.06	0.10	−0.04	0.08
Mid-piece	0.09	0.23	0.00	0.23	-0.09	0.11	0.20	0.16	−0.04	−0.08
Tail	−0.02	−0.13	−0.07	−0.13	0.09	0.27*	0.28*	0.25*	0.06	0.21
Total	0.15	0.28*	-0.16	0.28*	0.09	0.06	0.21	0.23	−0.05	0.14
HOST	0.003	−0.07	0.09	−0.07	−0.08	−0.18	−0.14	−0.14	0.02	−0.09
AI	−0.09	−0.16	−0.01	−0.16	−0.09	−0.14	−0.10	−0.10	0.15	0.01

E.Volume=Ejaculate volume, MA=Mass activity, IPM=Initial progressive motility, CON=Sperm concentration, NESC=Non-eosinophlic spermatozoa count, Head=Head abnormality, Mid-piece=Mid-piece abnormality, Tail=Tail abnormality, Total=Total abnormality, HOST= Hypo-osmotic swelling test, AI=Acrosomal integrity; SA=Sexual aggressiveness, RT=Reaction time, DT=Dismounting time, TTTM=Total time taken in mounts, PES=Penile erection score, PPS=Penile protrusion score, ETS=Ejaculatory thrust score, MAS=Mating ability score, LS=Libido score, SBS=Sexual behavior score.

** p<0.01,

* p<0.05

The SA showed highly significant (p<0.01) positive correlation with LS, SBS and non-significant negative correlation with RT and TTTM. The result indicates that the bulls showing higher SA normally better in libido. The results are in similar line as reported by Kumar [6] in Sahiwal bulls. RT had positive significant correlation (p<0.01) with TTTM and negative significant (p<0.01) correlation with LS and SBS, which is similar with the finding reported by Kumar [6] in Sahiwal bulls. In case of higher RT, the bulls usually take higher total time in mounts and shows poor libido. DT had positive significant correlation (p<0.05) with PES, PPS, MAS, SBS, and non-significant positive correlation with TTTM, ETS, and LS, which indicates the bulls taking more time to dismount, shows better penile erection, penile protrusion, mating ability, and SBS. TTTM had negative significant (p<0.01) correlation with LS and SBS. PES had positive significant (p<0.01) correlation with ETS, MAS, SBS, and LS (p<0.05). PPS showed positive significant (p<0.01) correlation with ETS, MAS, and SBS. ETS showed positive significant (p<0.01) correlation with MAS and SBS. The results predict that in case of better penile erection, penile protrusion, ejaculatory thrust, bulls show better LS, MAS, and SBS. MAS and LS both showed positive significant (p<0.01) correlation with SBS, whereas LS had a significant negative correlation (p<0.001) with RT. Assessment of sexual behavior is crucial to BSE of bulls. Understanding sexual behavior and its relationship with semen quality will facilitate identification of problem breeding bulls thereby minimizing the chance of inclusion of such bulls in the breeding program. RT, LS, and SBS are the important parameters to assess the sexual behavior of the bulls.

The results of relationship of different seminal attributes of Sahiwal bulls depict that ejaculate volume had positive significant (p<0.05) correlation with IPM, SCON, head abnormality, total abnormality, HOST, AI as well as non-significant positive correlation with MA, NESC, and pH. MA had positive significant (p<0.01) correlation with IPM, SCON, NESC, HOST, AI, and CYBR-14. The results are in consonance with the findings reported in crossbred bulls [7] and in Sahiwal bulls [8,9]. The initial progressive motility (IPM) had positive significant (p<0.01) correlation with NESC, HOST, AI. The results of the present study were in agreement with some previous work on cattle [4,10] The correlation that was recorded between IPM, viability, AI and HOST was expected since they are all related to plasma membrane integrity [11]. The semen samples are showing higher IPM, are also showing high SCON, NESC, HOST, and AI, as well as low mid-piece abnormality in the semen [12]. SCON showed positive significant (p<0.01) correlation with NESC and HOST also non-significant negative correlation with a morphological abnormality. The results are in agreement with reported by Patel *et al*. [7] in crossbred bulls. NESC had a positive significant correlation (p<0.01) with HOST and AI also negative significant (p<0.05) correlation with a total abnormality. The results obtained in the present study are in agreement with the reports in Sahiwal bulls [6,9] and in Murrah buffalo bulls [13] and in Ram [14] and Bhakat [15] in crossbred and Murrah bulls. Head abnormality showed positive significant (p<0.05) correlation with mid-piece abnormality and total abnormality. Mid-piece abnormality had significant (p<0.05) positive correlation with tail and total abnormality and negative correlation with IPM. The finding was similar with earlier reported in Murrah buffalo bulls [16] HOST had positive significant (p<0.01) correlation with AI which indicates, semen samples showing higher HOST positive spermatozoa then there will be more acrosomal integrity and livability in the spermatozoa. The results revealed that increasing sperm abnormalities tended to be associated with declining MA, IPM, HOST, and AI in the semen. Abnormality may affect any part of the spermatozoa, if a tail abnormality is increasing it is likely to adversely affect motility function of the spermatozoa. Whereas in case of increase in head abnormality it may interfere with acrosome status of the spermatozoa. In case of any defect in plasma membrane or body of the spermatozoa, it may result in impaired plasma membrane integrity of sperms. Effective management of breeding bulls and semen collection by the skilled collector will ensure desirable sexual behavior associated with better semen quality in bull stations thereby augmenting quality germplasm production.

The correlation coefficients between seminal attributes and sexual behavior parameter depicted that MA and IPM both had negative significant (p<0.05) correlation with RT and TTTM which indicates if more RT then there are poor MA and IPM of semen. Similar reports regarding MA and RT was reported by Elrabie [17]. SCON had positive significant (p<0.05) correlation with PPS, which indicates bulls have better penile protrusion have better SCON. Likewise, head abnormalities had positive significant (p<0.01) correlation with RT and TTTM, which predict that if more RT and TTTM then semen have more head abnormality in semen.

Sahiwal bulls are sexually sluggish and total time required to make breeding bulls donate quality ejaculates is more in indigenous bulls. It is, therefore, necessary to study the sexual behavior of individual bulls critically to take corrective measures for successful collection of semen. The individual preference of the bulls also needs to be studied for maximizing harvest of quality ejaculates as some bulls prefer false mount and some other bulls do not require false mount if they are restrained properly in the semen collection yard. Sometimes in the presence of dominant and adult bulls, the young bulls shows submissive behavior i.e., a form of animal behavior in which one individual attempts through appeasement displays to avoid injury by a dominant member of its own species and may results in reluctance of MO to the dummy. This behavior is more prominent in indigenous dairy bulls. The temperature of artificial vagina, timing of presentation of AV to the penis is very crucial in case of indigenous bulls as a fraction of a second little delay in grabbing the penis for diverting to AV; the bull will dismount early and become reluctant to mount again with a long refractory period. Semen quality and quantity is related with the sexual behavior of bull and specifically MA and volume parameters are more closely related with sexual behavior and ejaculatory thrust. As we are well aware that RT and ejaculatory thrust is important as it is highly correlated with quality as well as the quantity of semen. The standards of SBS and semen quality in comparison to crossbred bulls are lower in indigenous bulls, which reflects that, there is a need of involvement of expert technical persons for semen collection based on the individual preference of the indigenous bulls. Sufficient time should be given for sexual preparation of bulls starting from training of the bulls for semen collection, as it is a learning process. Overview of the results revealed the importance of sexual behavior and its relationship with semen quality to design appropriate management strategies to maximize quality semen production from the individual bulls.

## Conclusion

The correlation of various sexual behavior and semen quality parameters reflected the importance of sexual behavior in predicting the quality of the semen and their future utility. The overview of the results depicted the need of greater emphasis on management interventions to improve sexual behavior particularly SA and RT, which will be helpful in increasing ejaculate volume and total output of spermatozoa, which will lead to increase in production of frozen semen doses required for breed improvement program. The collection of semen should be based on the individual animal’s sexual behavior and preferences to harvest maximum sperm per ejaculate. The relationship of sexual behavior with semen quality will be helpful in analyzing the behavior of individual bull to provide appropriate management interventions to maximize the semen production of bulls by incorporating those in routine management practices in semen banks. Besides that sexual behavior and semen quality are the important criteria for declaring a bull satisfactory breeding bull during BSE of a male at sexual maturity. It will be helpful in reduction of rearing cost by rejecting the bulls which are not satisfactory in BSE.

## Authors’ Contributions

MB and TKM have designed the study. SS carried out the laboratory work and analysis of data. MB, TKM, AKG and AKC supervised the research and helped in drafting of the manuscript. AK and PS helped with the laboratory work. All authors read and approved the final manuscript.
